# Dr. William H. Bailey

**Published:** 1898-08

**Authors:** 


Dr. William H. Bailey, of Albany, died at his home in that city June 24, 1898, aged 72 years. He was born December 28, 1825, at Bethlehem, N. Y., and was the son of Dr. Solomon Bailey. After receiving an academic education he entered Albany Medical College, from which he received the doctorate degree in 1853. He began practice at Utica, but in the following year removed to Albany, where he continued in practice until his final illness. He was a member of the Medical Society of the State of New York, of which he was secretary from 1865 to 1875, and president in 1880. He was also a member of several other medical societies. He was an able physician, respected by a large community and died lamented by profession, citizens and family.

